# Supplementary material for: Compact inverse designed vertical coupler with bottom reflector for sub-decibel fiber-to-chip coupling on silicon on insulator platform
Source: Sci Rep. 2025 Jan 23;15:2925. doi: 10.1038/s41598-025-86161-1 (PMC11757707; doi:10.1038/s41598-025-86161-1)
Supplement: Supplementary file 1 — Supplementary Information. [file 41598_2025_86161_MOESM1_ESM.pdf]

Supporting Information: Compact Inversely-Designed Vertical Coupler with Bottom Reflector for Sub-Decibel Fiber-to-Chip Coupling on Silicon-on-Insulator Platform

Shiang-Yu Huang*,† and Stefanie Barz*,†,‡

†*Institute for Functional Matter and Quantum Technologies, University of Stuttgart, 70569 Stuttgart, Germany*

‡*Center for Integrated Quantum Science and Technology (IQST), University of Stuttgart, 70569 Stuttgart, Germany*

E-mail: shiang-yu.huang@fmq.uni-stuttgart.de; stefanie.barz@fmq.uni-stuttgart.de

Simulation of the final design after removing the bottom reflector

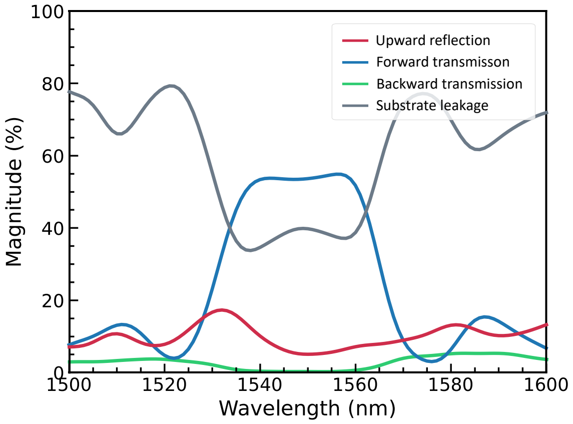

Figure S1: Forward (blue) and backward (green) transmission, upward reflection (red) and substrate leakage (grey) with respect to the wavelength after removing the bottom reflector from the final design.

After we acquire the design of the vertical coupler produced using topology optimization incorporating the bottom reflector, the reflector is removed from the coupler to study the effect it exerts. In the three-dimensional FDTD simulation, the Gaussian light source with y-polarization is positioned at the center of the coupler and injected from the top. The thickness of the buried oxide layer is still set to be 2 µm to have a fair comparison with the original design with a bottom reflector. The simulation is then performed within the wavelength range of 1500 nm to 1600 nm with a resolution of 1 nm.

After omitting the bottom reflector, the substrate leakage increases drastically to around 40% in the range of 1530 nm to 1560 nm (solid grey line in Fig. S1) while the upward reflection back to the optical fiber and the backward transmission remain relatively low (solid red and green lines in Fig. S1, respectively). Due to the massive loss towards the substrate, the forward transmission that correlates to the coupling efficiency of the coupler is reduced to around 55% at 1550 nm (solid blue line in Fig. S1). Therefore, the bottom reflector is important for improving the overall coupling efficiency since it enhances the directionality of the topology-optimized vertical coupler.

Exclude the bottom reflector in the topology optimization

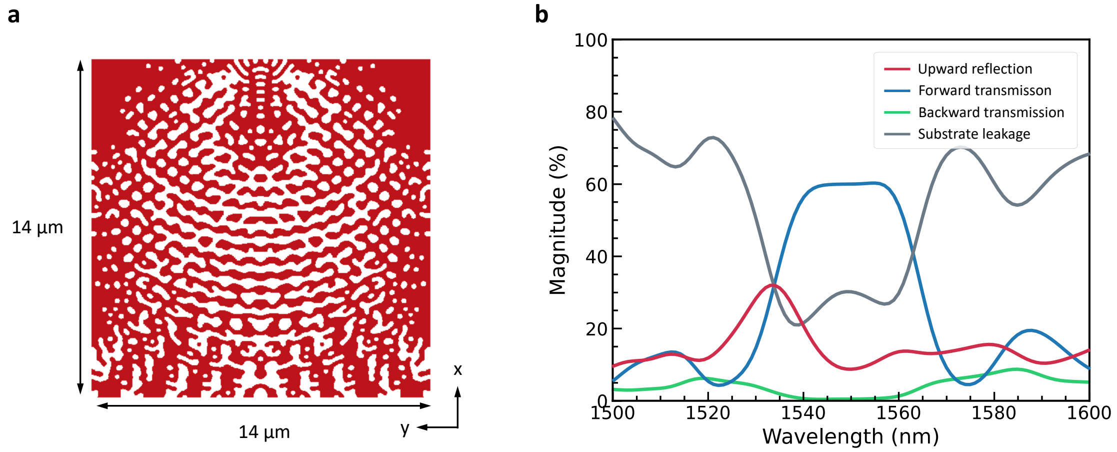

Figure S2: (a) The refractive index distribution of the design delivered by the optimization excluding the bottom reflector. The maroon (white) color corresponds to the reflective index of silicon (SiO_2). (b) Forward (blue) and backward (green) transmission, upward reflection (red) and substrate leakage (grey) with respect to the wavelength of the design produced by the optimization excluding the bottom mirror.

We also apply the topology optimization where the bottom reflector is excluded in the design of a vertical coupler. The settings regarding the simulation and optimization remain the same as the descriptions outlined in Method section in the main text. The total iteration number of the optimization in this case is 393 and the duration of the optimization is roughly 12 days. The resulting design (Fig. S2(a)) shares a similar feature with the design delivered by the optimization incorporating a bottom reflector. The simulated coupling efficiency is -2.23 dB (59.8%) at the wavelength of 1550 nm.

Topology-optimized vertical coupler delivered using a different initial condition

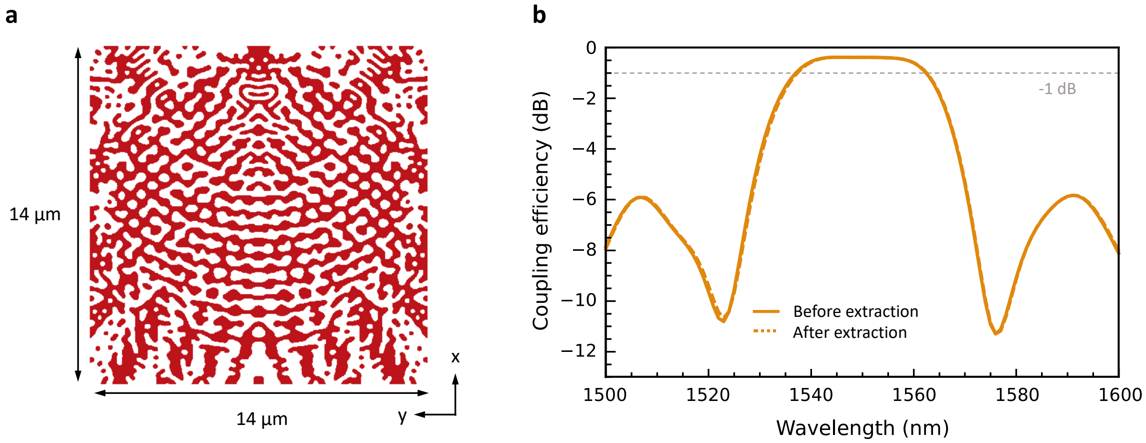

Figure S3: (a) The refractive index distribution of the design delivered by the optimization beginning with the mixture initial condition. The maroon (white) color corresponds to the reflective index of silicon (SiO_2). (b) Simulated coupling efficiency of the island-based topology-optimized vertical coupler with respect to the wavelength before (solid line) and after extraction (dashed line).

As the adjoint method is a gradient-descent method, the initial condition of the design region may significantly influence the final output of the topology optimization. Here we demonstrate the result of the topology optimization using the "mixture" initial condition. In this case, a pseudo material of which the refractive index is the average of silicon and SiO2 is set in the design region initially. Other settings regarding the simulation and optimization remain the same as the descriptions outlined in the Method section in the main text. After the optimization is completed, the final structure (Fig. S3 (a)) is also simulated subsequently with a broader wavelength range and higher spectrum resolution.

The total iteration number of the optimization is 246, which takes around 10 days. The simulated coupling efficiency of the "island-based" coupler across the wavelength from 1500 nm to 1600 nm shows comparable performance with the "hole-based" coupler shown in the main text. At the wavelength of 1550 nm, the coupling efficiency is -0.374 dB (91.7%). The structure of the coupler is also extracted and imported into the FDTD simulation environ-

ment for another subsequent simulation. The simulation result shows that the performance of the device remains almost unaffected (Fig. S3 (b)).
